# Behavioural factors associated with diarrhea among adults over 18 years of age in Beijing, China

**DOI:** 10.1186/1471-2458-14-451

**Published:** 2014-05-13

**Authors:** Chunna Ma, Shuangsheng Wu, Peng Yang, Haiyue Li, Song Tang, Quanyi Wang

**Affiliations:** 1 Beijing Key Laboratory of Diagnostic and Traceability Technologies for Food Poisoning, Beijing Center for Disease Prevention and Control, Beijing 100013, China; 2 The Institute of Environmental and Human Health, Texas Tech University, Lubbock, TX 79416, USA

**Keywords:** Diarrhea, Behavior, Influence factor

## Abstract

**Background:**

To date, a large proportion of people still suffer from diarrhea diseases. In addition to the burden of diarrhea, there are substantial social and economic costs caused by the high incidence of diarrheal diseases. Therefore, the purpose of this study was to explore the self-reported prevalence of diarrhea and associated risk factors of diarrhea among adults in Beijing, China.

**Methods:**

A multistage, stratified study based on cross-sectional data was performed using randomized and systematic sampling, recruiting 12,936 adults over 18 years of age in Beijing. All adults were requested to complete a questionnaire, including information such as demographic characteristics, incidence of diarrhea, and behaviors related to the diarrhea.

**Results:**

The self-reported prevalence of diarrhea was 17.5% during the last year prior to the survey. Six behavioral factors were significantly associated with diarrhea in our study including: (1) washing hands before meals and after defecation (Adjusted Odds Ratio (AOR) 0.707, 95% CI 0.597 ~ 0.837), (2) washing hands with soap and running water (AOR 0.872, 95% CI 0.786 ~ 0.967), (3) consuming raw seafood (AOR 1.285, 95% CI 1.138 ~ 1.450), (4) using the same chopping block and knife when processing raw and cooked food (AOR 1.375, 95% CI 1.225 ~ 1.542), **(**5) using the same chopsticks to handle raw and cooked food (AOR1.149, 95% CI 1.041 ~ 1.268), and (6) regularly participating in physical exercise (AOR 0.719, 95% CI 0.651 ~ 0.793).

**Conclusion:**

Good health habits, good eating habits, and regular exercise can prevent the episodes of diarrhea, and thus decrease the potential for disease occurrence.

## Background

Diarrheal diseases are common world-wide and can induce a broad spectrum of symptoms ranging from mild discomfort to dehydration or death if treatment is not administered. In the International Statistical Classification of Diseases and Related Health Problems 10th Revision (ICD-10), different forms of diarrhea are listed including infective neonatal diarrhea, unspecified diarrhea, functional diarrhea, non-infective neonatal diarrhea, and irritable bowel syndrome with diarrhea. Diarrhea can be caused by bacterial infection, viral infection, parasites, drugs, psychological factors, allergies, etc. A systematic analysis for the Global Burden of Disease (GBD) indicated that there are ten aetiologies for diarrhea in the cause list for 2010 [1].

Episodes of diarrhea illness can cause serious complications, such as dehydration, acidosis, and viral myocarditis, particularly in more vulnerable groups including children, elderly, or those with chronic disease [2]. GBD data from World Health Organization (WHO) showed that diarrheal disease is the fifth most common cause of death, which accounted for 3.7% (2.2 million) of all deaths in 2004 [3]. In 2010, 1.4 million people, across all ages, died from diarrheal diseases, and the age-standardized death rate was 20.9 per 100,000, which has fallen 49% between the years of 1990 and 2010 [1]. Although death rates from diarrheal diseases have been dramatically declining over the past twenty years, a large proportion of people still suffer from such diseases. In addition to the burden of disease, a large proportion of people still suffer from such diseases. In addition to the burden of disease, there are also substantial social and economic costs caused by the high incidence of diarrheal diseases [4-6]. Furthermore, people with diarrhea generally have a lower health-related quality of life, increased absenteeism from work or school, and reduced social interaction.

To date, there are no effective vaccinations against the various causes of diarrhea disease. Therefore, decreasing the morbidity is the most fundamental and effective approach to minimize the impact of diarrhea. A variety of risk factors have been identified in previous studies, including food contaminate, distance between the septic tank and well water, drink contaminate, household behaviors, restaurant sanitation, and environmental hygiene [7-12]. Medical examination and treatment including gastrointestinal endoscopy and early use of a pacifier have been found to lead to diarrhea [13,14]. In addition, total monthly household income is an influence factor for diarrhea [12]. The contribution of individual factors influencing the onset of diarrhea varies in different areas or regions due to cultural differences, eating habits and behaviors, economic status, among others. In order to design effective prevention strategies and interventions against episodes of diarrhea among the adults in Beijing, identifying factors, especially risk behaviors, which contributed most to diarrhea is very important. Few large-scale population surveys have been conducted to identify the prevalence of diarrhea and characterize the risk factors associated with diarrhea in adults of Beijing. Therefore, the aim of the present study was to investigate the prevalence of diarrhea relating to influential factors of behaviors among adults in Beijing, China.

## Methods

### Subjects

The target population was adults over 18 years of age in Beijing, China. The respondents were classified into 20 subgroups according to living area (urban or suburban), gender (male or female) and age group (18 to 29, 30 to 39, 40 to 49, 50 to 59, and 60 and above). The following formula was used to estimate the sample size in each subgroup: n = (t^2^pq/d^2^)*deff, where t = 1.96 (Type I error), P = 50% (we could not get the reporting rate of diarrhea among subjects, thus P is equal to 50 to attain maximum sample size), q = 1 – p, d = 0.1p (permissive error) and a hypothesis of design effect (*deff*) of 1.5. Assuming that no-answer rate is 15%, then, a sample size of 13,248 questionnaires was calculated to obtain.

Beijing is the capital of China, has a population greater than 20 million people, and is divided into 16 districts, including 6 urban and 10 suburban districts. The survey was undertaken in six randomly selected districts, including three urban districts and three suburban districts. Participants were recruited using a multi-stage stratified sampling method in each district. Firstly, five towns/streets per district were randomly selected. Secondly, five villages/communities in each of the towns/streets were randomly selected. Thirdly, 29–44 households per village/community were randomly selected for interviews. The investigators visited the households individually, and interviewed each adult within the households until 87–89 residents were investigated in each community/village.

### Data collection

A pre-test was conducted in different education levels and age groups to assess suitability with regard to study comprehension, language appropriateness, and duration. All investigators were trained in research and interviewing skills, content of questionnaire, and data quality. Before implementing the study, a letter which explained the objectives of the research was provided to all eligible participants. Then, questionnaires were carried out among those who gave verbal consent of participation. Respondents completed the questionnaire under the supervision of the trained investigators. Data in each of the questionnaires were checked carefully for incorrect or omitted items, and logistical errors by investigators.

### Variable specification

The questionnaire consisted of three parts, demographic information (gender, age, education level, occupation, and living area), diarrhea, and behaviors. Diarrhea was defined according to criteria used by the WHO: 3 or more loose or liquid stools per day, or more frequently than is normal for the individual [15]. People were queried as to whether they have had diarrhea during the last year prior to the survey. They also answered questions regarding whether they washed hands before meals and after defecation for the day prior to diarrhea (every time, most of time, sometimes, almost not, or never), and the type of hand washing (soap with running water or others). Other questions included if subjects eat raw seafood or freshwater products (yes or no), use the same chopping block and knife when processing raw food and cooked food (yes or no), and use the same chopsticks to pick up raw food and cooked food when eating instant-boiled mutton slices (yes or no). Additionally, questions were asked if choice of restaurant was based on sanitary conditions; if subjects participate in regular physical exercise (more than three times every week, more than 30 minutes every time and medium to high intensity of exercise; yes or no).

### Ethics Statement

This study was approved by the Institutional Review Board and Human Research Ethics Committee of Beijing Center for Disease Prevention and Control. At the beginning of each interview, the agreement and verbal consent of the interviewee was obtained. Anonymity of the participants was guaranteed.

### Statistical analysis

Descriptive statistics (frequencies and proportions) were calculated for all items in the questionnaire. Weighted analysis was conducted to calculate the total incidence of diarrhea,the data were weighted for age and gender. As described previously [16,17], the univariate logistic regression analysis was conducted to assess the factors that contributed to diarrhea. Multivariate logistic regressions adjusted for age groups, education level, and living area, were used to test the individual factors that led to diarrhea. A probability of *P* ≤ 0.05 was considered statistically significant. All statistical tests were two-sided. The data were processed, and statistical analysis was performed with SPSS 16.0 software (SPSS Inc., Chicago, IL, USA).

## Results

The socio-demographic characteristics of the participants are illustrated in Table 1. Of the 12,936 respondents, the average age was 44.81 years (95% CI 44.81 ± 0.25), approximately 48% were male and 52% were female. The number of respondents at different age groups ranged from 2,514 to 2,683. More than 86% of respondents’ education level was junior high school or above. Of all respondents, 2,265 (17.5%) suffered from diarrhea during the last year. The self-reported rate of diarrhea was significantly different among age groups, education levels, and living areas (*P* < 0.05). In this study, the communities and villages were not selected with probability proportional according to population size, and the sample might not be representative of the underlying population. Therefore, weighted analysis was conducted to calculate the total incidence of diarrhea, which was17.9% after weighted for age and gender.

**Table 1:** Demographic characteristics of the participants

**Demographic characteristics**	**Number of people answering questions**	**Number of people reporting diarrhea (%)**	**Bivariate analysis (χ2 test)**	***P*-value**
**Gender**				
Male	6246	1103(17.7)	0.188	0.664
Female	6690	1162(17.4)		
**Age, years**				
18~	2683	534(19.9)	15.953	0.003
30~	2526	435(17.2)		
40~	2582	424(16.4)		
50~	2631	427(16.2)		
60~	2514	445(17.7)		
**Education level**				
Illiterate or literacy seldom	385	84(21.8)	24.115	<0.001
Primary school	1404	290(20.7)		
Junior high school	3638	648(17.8)		
Senior high school	3674	573(15.6)		
College and above	3835	670(17.5)		
**Occupation**				
Student	431	85(19.7)	15.076	0.129
Farmer	3772	670(17.8)		
Workers engaged in production or transportation	329	64(19.5)		
Enterprise or company staff	2111	353(16.7)		
Migrant workers	421	74(17.6)		
Commercial services personnel	1071	212(19.8)		
Civil servants or public institutions personnel	1228	199(16.2)		
Medical personnel	323	70(21.7)		
Retirees	2034	330(16.2)		
Housewife or jobseekers	981	167(17.0)		
Others	235	41(17.4)		
**Living area**				
Urban	6417	1079(16.8)	4.253	0.039
Rural	6519	1186(18.2)		
**Total**	12936	2265(17.5)		

The self-reported prevalence of diarrhea among people who wash their hands before meals and after defecation (16.9%) is significantly lower than people who do not wash their hands in the same circumstances (24.1%) (*P* < 0.05) (Table 2). Approximately, 16% of people who wash their hands with soap and running water reported suffering from diarrhea, and the self-reported rates were significantly higher for people with inappropriate manners of hand-washing (19.4%) (*P* < 0.05). Moreover, there was a significant difference for those who self-reported the prevalence of diarrhea between the group of eating raw seafood (21.3%) and the group of never eating those foods (16.8%) (*P* < 0.05). Those who use the same chopping block and knife when processing raw and cooked foods self-reported a greater prevalence compared to those who use different chopping blocks and knives (*P* < 0.05). Additionally, those who use the same chopsticks to pick up raw food and cooked food when eating instant-boiled mutton slices self-reported a greater prevalence rate of diarrhea than those using separated (*P* < 0.05). However, there was no statistical significance found in self-reported diarrhea prevalence rates between people choosing to eat at restaurants based on their sanitary conditions (17.0%) and those who do not take sanitary conditions into account (18.3%) (*P* > 0.05). Meanwhile, self-reported prevalence rates of diarrhea among people being used to regular physical exercise (15.3%) was significantly lower than those with a more sedentary lifestyle (21.0%) (*P* < 0.05).

**Table 2:** Factors in behaviors that contributed to diarrhea

**Behaviors**	**Number of reporting people**	**Number of people reporting diarrhea (%)**	**OR**	***95%CI***	***Wald***	***P *** **value**
**Washing hands before meals and after defecation for the day**						
Sometimes, almost not, never	1085	261(24.1)	1.000	Reference	34.663	<0.001
Every time, most of time	11851	2004(16.9)	0.643	0.555 ~ 0.744		
**The type of hand washing**						
Others	5652	1098(19.4)	1.000	Reference	25.486	<0.001
Soap and running water	7284	1167(16.0)	0.791	0.723 ~ 0.867		
**Eating raw seafood or freshwater products**						
No	10668	1787(16.8)	1.000	Reference	24.105	<0.001
Yes	2268	478(21.1)	1.372	1.185 ~ 1.486		
**Using the same chopping block and knife at the time of processing raw food and cooked food**						
No	10088	1620(16.1)	1.000	Reference	66.098	<0.001
Yes	2848	645(22.6)	1.530	1.381 ~ 1.696		
**Using the same chopsticks to pick up raw food and cooked food when eating instant-boiled mutton slices**						
No	6522	1035(15.9)	1.000	Reference	24.431	<0.001
Yes	6414	1230(19.2)	1.258	1.148 ~ 1.378		
**If choosing to eat at restaurants or not based on their sanitary conditions**						
Sometimes, almost not, never	5437	989(18.2)	1.000	Reference	3.01	0.083
Every time, most of time	7499	1276(17.0)	0.922	0.842 ~ 1.011		
**Regularly participating in physical exercise**						
No	5078	1062(20.9)	1.000	Reference	61.137	<0.001
Yes	6576	1007(15.3)	0.684	0.622 ~ 0.752		

Six factors were significantly associated with diarrhea after adjustment for age, education level, and living area (*P* < 0.05; Table 3). They were: (1) hand-washing before meals and after defecation (AOR 0.707, 95% CI 0.597 ~ 0.837), (2) hand-washing with soap and running water (AOR 0.872, 95% CI 0.786 ~ 0.967), (3) eating raw seafood or freshwater products (AOR 1.285, 95% CI 1.138 ~ 1.450), (4) using the same chopping block and knife when processing raw and cooked food (AOR 1.375, 95% CI 1.225 ~ 1.542), (5) using the same chopsticks to pick up raw and cooked food when eating instant-boiled mutton slices (AOR1.149, 95% CI 1.041 ~ 1.268), and (6) regularly participating in physical exercise (AOR 0.719, 95% CI 0.651 ~ 0.793).

**Table 3:** Multivariate logistic regression analysis model contributing behaviors factors of diarrhea

**Influence factors in behaviors**	***β***	***Wald *** **value**	***P *** **value**	Adjusted^* ^ ***OR *** **(95% *CI*)**
Washing hand before meals and after defecation	-0.35	16.185	<0.001	0.707(0.597 ~ 0.837)
Washing hand with soap and running water	-0.14	6.735	0.009	0.872(0.786 ~ 0.967)
Eating raw seafood or freshwater products	0.251	16.409	<0.001	1.285(1.138 ~ 1.450)
Using the same chopping block and knife at the time when processing raw and cooked food	-0.32	29.317	<0.001	1.375(1.225 ~ 1.542)
Using the same chopsticks to pick up raw food and cooked food when eating instant-boiled mutton slices	0.139	7.568	0.006	1.149(1.041 ~ 1.268)
Regularly participating in physical exercise	-0.33	45.555	<0.001	0.719(0.651 ~ 0.793)

*Adjusted for age group, education level, and living area.

## Discussion

In this study, the self-reported prevalence of diarrhea during the last year prior to the survey was 17.5% among adults in Beijing. A previous study, published in 2005, showed that the prevalence of reporting diarrhea in four weeks prior to interview were 6.5% in Australia, 7.9% in Canada, 3.6% in Ireland, and 7.8% in United States, with diarrhea being defined as more than 3 loose stools or bowel movements in any 24 h period in the study [2]. Similarly, Wheeler J G et al. found that the retrospective estimate of reported rate of diarrhea in the last month was 6.5% in England which was published in 1999 [18], and Feldman R A et al. reported it was 7.9% in Great Britain [19]. If the reporting rates of diarrhea in the past days in above studies are converted to one year, it might be higher than the results of the study presented here. The difference may partially be due to the change of behaviors and sanitary condition over time, and the diarrhea definition. However, we cannot rule out the possibility that the differences are caused by recall bias. Moreover, our result is different compared to Elaine et al. showing the prevalence of diarrhea was consistently higher in females, while it was lowest in person more than 65 years of age among Australia, Canada, Ireland, and United States and it was published in 2005 [2]. These differences might be associated with the variations in the cause of diarrhea as well as the behavior model of people between developed countries and developing countries.

Many types of pathogens, such as *Vibrio cholera, Shigella spp*, *Salmonella spp*, and rotavirus, multiply in the human gut, exit in excreta, and transition through the environment, which can ultimately cause diarrhea in new hosts [20,21]. Because pathogens of diarrheal diseases occur in feces, the prevention of fecal matter from polluting the environment is likely to be of great significance to public health [22]. Safe stool disposal and adequate hand washing, especially after defecation, are the key principal barriers to the transmission of enteric pathogens [21]. Hands can serve as a medium, which transmit pathogens to food and drink and ultimately to the mouths of susceptible individuals. This is supported by a systematic showing hand washing reduced diarrhea incidence with a median of 35% in five studies [23]. Similarly, another review demonstrated that washing hands with soap can reduce the risk of diarrheal diseases by 42% [20]. In the present study, the risk of suffering diarrhea among the subjects washing their hand before meals and after defecation reduce diarrhea incidence by 29%, which further confirmes previous findings in different countries.

A wide range of bacteria, viruses, and parasites have been involved in seafood-related outbreaks, which are reported globally [24]. The risk factor most commonly associated with infection is consumption of raw or undercooked seafood [24]. At least ten genera of bacterial pathogens have been involved in seafood-borne diseases [25]. Previous research shows that consumption of raw or undercooked seafood contaminated with *Vibrio parahaemolyticus* may result in development of acute gastroenteritis characterized by diarrhea, and this pathogen is a common cause of food-borne illness in Asia, including China, Japan, and Taiwan [26]. Kain K C et al. found that among *P. shigelloides-infected* patients, 29% acquired the infections locally in association with the consumption of seafood or untreated water or both [27]. This gives evidence that there is a significantly greater frequency of occurrence of diarrhea in people who eat raw seafood over people who do not, thus education should be implemented with regard to safe food preparation habits and dietary health habit.

It has been well established that improper practices of food preparation in consumer homes contribute to a substantial proportion of food-borne diseases [28,29]. For instance, bacteria, including *Salmonella spp* can be readily transferred to cutting boards and utensils during food preparation, which can cross-contaminate other foods if the boards and utensils are not cleaned [30]. It has been estimated that cross-contamination in the household kitchen resulting in food poisoning can be attributed to contamination from *Escherichia coli* O157:H7 (40%), *Campylobacter* (30%), and *Salmonell*a (20%) [11]. Our results are similar to previous findings showing that factors associated with a lower risk of self-reported diarrhea used separate chopping boards when processing raw meats and cooked meats (OR 0.803, 95% CI 0.648 **~** 0.994) or other raw and cooked foods (OR 0.741, 95% CI 0.599 **~** 0.919) [11].

There are some limitations in our study. The first and main limitation is recall bias. The study contents involved cases of diarrhea throughout a year prior to filling out the questionnaire. The information was collected retrospectively, and the interviewees may be likely to have forgotten about their high-risk behaviours. Secondly, some respondents suffered from diarrhea more than once, thus the prevalence rates may have been underestimated in our study. Thirdly, most of the risk factors in our regression model are associated with infective diarrhea, however, non-infective diarrhea caused by drugs, psychological factors, allergies, etc. also contributed to diarrhea of the respondents in last year. Hence, additional risk factors related to non-infective diarrhea should be taken into account in future studies. The results of this study provide evidence that more attention should be given to the issues regarding domestic kitchen hygiene, especially food handling, preparation, and storage practices, in order to effectively and efficiently reduce the prevalence of diarrhea in the developing world.

## Conclusions

This is a large-scale population surveys to identify the prevalence of diarrhea and explore the risk factors associated with diarrhea in adults in Beijing. The self-reported prevalence of diarrhea was lower than some other countries. The results suggest that good health habits, good eating habits, and regular exercise can prevent the episodes of diarrhea, and thus decrease the potential for disease occurrence.

## Competing interests

The authors declare that they have no competing interests.

## Authors’ contributions

MC, WS, TS and WQ designed the study. MC, WS, YP and LH performed the data collection. MC analyzed the data and wrote the manuscript. All authors have read and approved the final version of the manuscript.

## Pre-publication history

The pre-publication history for this paper can be accessed here:

http://www.biomedcentral.com/1471-2458/14/451/prepub
